# Characterization of Potential Virulence Factors of *Vibrio mimicus* Isolated from Fishery Products and Water

**DOI:** 10.1155/2021/8397930

**Published:** 2021-02-10

**Authors:** M. F. Hernández-Robles, I. Natividad-Bonifacio, A. K. Álvarez-Contreras, J. J. Tercero-Alburo, E. I. Quiñones-Ramírez, C. Vázquez-Salinas

**Affiliations:** ^1^Departamento de Microbiología, Escuela Nacional de Ciencias Biológicas, Instituto Politécnico Nacional,Prolongación Carpio y Plan de Ayala S/N, Ciudad de México, Colonia Santo Tomas,CP. 11340, Mexico; ^2^Departamento de Biotecnología, División de Ciencias Biológicas y de la Salud, Universidad Autónoma Metropolitana-Iztapalapa, Avenida San Rafael Atlixco 186, Ciudad de México, Colonia Vicentina,C.P. 09340, Mexico

## Abstract

*Vibrio mimicus* is a Gram-negative bacterium that is closely related to *V. cholerae* and causes gastroenteritis in humans due to contaminated fish consumption and seafood. This bacterium was isolated and identified from 238 analyzed samples of sea water, oysters, and fish. Twenty strains were identified as *V. mimicus* according to amplification of the *vmhA* gene, which is useful as a marker of identification of the species. The production of lipases, proteases, and nucleases was detected; 45% of the strains were able to produce thermonucleases and 40% were capable of producing hydroxamate-type siderophores, and the fragment of the *iuT* gene was amplified in all of the *V. mimicus* strains. Seventy-five percent of *V. mimicus* strains showed cytopathic effect on Chinese hamster ovary (CHO) cells and destruction of the monolayer, and 100% of the strains were adherent on the HEp-2 cell line with an aggregative adherence pattern. The presence of virulence factors in *V. mimicus* strains obtained from fishery products suggests that another member of the *Vibrio* genus could represent a risk to the consumer due to production of different metabolites that allows it to subsist in the host.

## 1. Introduction

*Vibrio mimicus* is a Gram-negative bacterium that is closely related to *V. cholerae* and causes gastroenteritis characterized by diarrhea, nausea, vomiting, abdominal pain, and fever due to contaminated fish consumption and seafood [1, 2]. The infective dose is unknown, but it is believed to be the same as that of *V. cholerae*, ranging from 10^4^ to 10^6^ cells [3]. This bacterium has been isolated from water and a variety of fishery products, such as oysters, sea turtle eggs, shrimp, crab, and fish [4].

The mechanisms of the pathogenicity of *V. mimicus* are unknown; however, it has been reported that *V. mimicus* produces several virulence factors, including adhesins, hemolysins, and various types of proteases (collagenases and metalloproteases), siderophores, cytolysins, lipases, and DNAses [5, 6]. This bacterium produces a heat-labile cytolytic/hemolytic toxin called *Vibrio mimicus* hemolysin (VMH) encoded in *vmhA* found in environment and clinical strains [7, 8]. Thus, *vmhA* gene is a useful marker of identification of this species [7].

It has been reported that *V. mimicus* shares some genotypic characteristics with *V. cholerae* such as the *ctx*AB operon which encodes choleric toxin whose gene is found in the genome of bacteriophage *CTXΦ* and infects *V. cholerae*, indicating horizontal transfer of this phage between *V. cholerae* and *V. mimicus* [9, 10].

There are several reports on infections due to *V. mimicus* in other countries, suggesting strains must harbor genes that can cause infections due to consuming raw or undercooked fishery products; however, there are no studies on the virulence markers that are harbored in wild-type strains. We showed the presence of some virulence factors in strains isolated from environmental samples.

## 2. Materials and Methods

A total of 238 samples were collected from 12 different sites (11 oysters, 11 fish, and 12 sea water samples per month) in the Pueblo Viejo Lagoon, Veracruz, México, for seven months (June to December 2017).

The species captured were white mullet (*Mugil curema*) and American oyster (*Crassostrea virginica*). Fish and oysters samples were transported in individually labeled and sealed plastic bags. Seawater samples were collected in labeled plastic jars. The samples were cooled at 4°C immediately after collection and transported to the laboratory for analysis.

### 2.1. Isolation and Phenotypical Identification of *V. mimicus*

*V. mimicus* was isolated and identified as described in the Bacteriological Analytical Manual of the Food and Drug Administration [11]. Each sample was homogenized (Stomacher® 400 *Circulator*), 50 g was placed in 450 mL flasks containing alkaline peptone water (APW, pH 8.8) to obtain duplicated dilutions from 1 : 10, 1 : 100, and 1 : 1000 and incubated at 37°C and 42°C for 6–24 h. In water samples, 25 mL was homogenized in 225 ml of alkaline peptone water and incubated for at least 6 h at 37°C [12]. Each dilution was streaked onto thiosulfate-citrate-bile salts-sucrose agar plates and incubated at 37°C for 18–24 h; three suspected *V. mimicus* colonies were selected from each plate. Halophilism tests were performed on tryptone agar containing 0, 3, 6, 8, and 10% NaCl. The API 20E system (BioMerieux™) and *vmhA* amplification was used for identification.

Control strains used in this study were *Vibrio mimicus* ATCC 33653, *Vibrio vulnificus* ATCC 29307, *Vibrio cholerae* O1 Ogawa, *Vibrio cholerae O1 Inaba*, and *Vibrio cholerae* no O1 CLBM-ENCB.

### 2.2. Determination of the Proteolytic, Lipolytic, and Hemolytic Activities as well as Nuclease and Thermonuclease Production

Cells were grown overnight in tryptic soy agar with 2% NaCl at 37°C and spot-inoculated onto the plated assay media as described by García and Landgraf [13]. Protease activity was determined using casein (2% skim milk) as substrate, and lipase activity was assessed in nutrient basal agar containing 10% (v/v) egg yolk emulsion. To detect hemolysis, strains were streaked on blood agar with 5% sheep erythrocytes and blood agar with 5% rabbit erythrocytes. To assess the presence of nucleases, 200 *µ*L of an overnight culture of *V. mimicus* was inoculated on DNAse agar [14]. For the thermonuclease assay, a fresh culture of *V. mimicus* was placed in a 100°C water bath for 10 min, and then, 200 *µ*L of this culture was inoculated on wells of agar DNA with toluidine blue and incubated at 37°C for 6 h [15].

### 2.3. Presence of Siderophores

*V. mimicus* strains were incubated at 37°C for 18–24 h on nutrient broth, and afterwards, they were inoculated on chrome azurol S (CAS) agar [16]. *V*. *mimicus* ATCC 33653 was used as a positive control.

### 2.4. Assessment of the Cytotoxic Effect on Chinese Hamster Ovary Cells

*V. mimicus* strains were inoculated in AKI broth (peptone 15 g/L, yeast extract 4 g/L, and sodium chloride 5 g/L pH 7.4) and incubated for 18 h with shaking (5 g) at 37°C. The culture was centrifuged at 500 g for 10 min, and the supernatant was filtered using a 0.22-*µ*m pore membrane. *V. cholerae* O1 Serotype Ogawa and Inaba were used as positive controls, and AKI broth without inoculum was used as a negative control. A total of 200 *µ*L of CHO suspension in F12 media with 15% fetal bovine serum (SFB) was placed on each of the 96 wells of an ELISA plate. The plates were incubated at 37°C in a 5% CO_2_ atmosphere until they reached 100% confluence. After the incubation time elapsed, media were discarded, and the plate was washed three times with sterile phosphate saline buffer (PBS). 100 *µ*L of 1 : 1, 1 : 3, 1 : 9, 1 : 27, 1 : 81, 1 : 253, 1 : 729, and 1 : 2187 dilutions per triplicate on F12 media of the filtrate of each one of the strains were added to each well and the plate was incubated at 35°C with 5% CO_2_. The plates were observed under an inverted microscope every 60 min until the observation of alterations of 50% of monolayer cells. Cytopathic and cytotoxic effects were positive when more than 50% of the cells showed destruction or alterations in their morphology [5].

### 2.5. Adherence Assay on HEp-2 Cell Line

Adherence assays were performed in human laryngeal carcinoma cell (HEp-2) monolayers grown on coverslips in 24-well microtiter plates and grown to confluence at 37°C in 5% CO_2_. Cell monolayers were inoculated in triplicate with 25 *μ*L of bacterial suspension. The plates were incubated at 37°C with a 5% CO_2_ atmosphere for 1.5 h, monolayers were then washed three times with PBS to remove nonadherent bacteria, and then the cells were fixed with methanol for 1 min and washed 3 times with sterile PBS and were Giemsa-stained for 20 min. The wells were washed with distilled water, dehydrated with acetone-xylol, and sealed with a drop of Permount resin. Cells were observed under a microscope at 100x. Adherence assay was positive when more than 40% of the cells had adherent bacteria [5].

### 2.6. Genetic Analysis

A Wizard Genomic DNA Purification Kit (Promega, Madison, WI, USA) was used to obtain DNA. The reaction mixture was prepared with 34.95 *μ*L of distilled water, 5 *μ*L of 10x buffer (200 mM Tris-HCl pH 8.4 and 500 mM KCl), 2.5 *μ*L of MgCl_2_ 50 mM, 0.25 *μ*L of a dNTP mixture (10 mM), 2.5 *μ*L of each primer (1 nM), 0.3 *μ*L of *Taq* polymerase (5 U/*μ*L), and 2 *μ*L of the DNA containing solution (approximately 100 ng) in a final volume of 50 *μ*L. PCR was performed on a MultiGene Gradient DNA Thermal Cycler MIDSCI with the primers and conditions reported in Tables 1 and 2. Obtained fragments were detected on agarose gels that were observed on Bio-Imagen Systems®. Images were digitalized with the MBE-IMG® (Mayor Science®) program.

## 3. Results and Discussion

**Figure 1:**
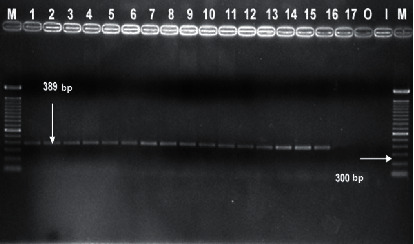
Electrophoresis gel of *vmhA* gene amplification product. Lanes (M): 100 bp molecular weight marker; lanes 1–15: isolated strains of *V. mimicus*; lane 16: *V. mimicus* ATCC 33653; lane 17: *Vibrio vulnificus* ATCC 29307; lane (O): *V. cholerae* O1 Ogawa; lane (I): *V. cholerae* O1 Inaba.

Of the 238 analyzed samples, a total of 1455 colonies with characteristics of the genus *Vibrio* were phenotypically identified, and only 20 were confirmed as *V. mimicus* (6 were isolated from sea water samples, 10, from oysters, and 4, from fish) by API 20E and genotypically by *vmhA* amplification (Figure 1). *V. mimicus* infections have been associated with gastroenteritis after seafood ingestion due to the fact that, in different countries, it is customary that oysters are to be eaten straight from the valves [18]. In fact, almost all reported cases are related to bivalves consumption as the source of direct contamination and/or turtle eggs as a cross-contamination [19, 20]. Additionally, fish is consumed without any thermic treatment like “ceviche,” a typical raw seafood dish where only lemon is added; these cultural traditions performed in holiday seasons are risk factors to contract diseases related to this bacterial genus.

All analyzed strains produced *β* hemolysis on sheep and rabbit erythrocytes (Table 3); Alam [21] and Beshiru [22] obtained 80% of hemolytic strains. Similarly, Miyoshi [23] reported that *V. mimicus* lyses horse, sheep, and human erythrocytes. The main hemolysin presence in this species is VMH, which showed 76% homology with the *hlyA* of *V. cholerae* el Tor; VMH is capable of forming pores on the cell surface and also promotes the production of cAMP, causing diarrhea [24, 25].

*vmhA* gene is considered a specific gene, and it is found in the wild-type and clinical strains [7]. Wei et al. [26] showed that gene amplification is correlated with other identification techniques as fatty acids profiles and 16S *DNAr*, *oriC*, *pyrH*, *recA*, and *rpoA* gene sequences comparison. In this study, *vmh* gene was amplified in all work strains (Table 3).

Regarding the enzymatic testing, all strains were positive for the production of lipases and proteases, and only 45% of the strains were positive for thermonuclease production. These results are similar to those reported by Alam [20], which showed that 95% of *V. mimicus* strains were positive for protease production. Beshiru [21] showed that 90% of their *V. mimicus* isolated were positive to protease activity. It has been reported that bacterial proteases are an extensive collection of enzymes that have important roles in pathogenicity, stress response, and cell viability [27–29].

All of the isolated strains in this study had lipolytic activity. These results are similar to those reported by Beshiru [22]. Davis [30] indicated that only 10% were positive for lipase activity at 48 hours, and Fiore [31] indicated that 95% of *V. mimicus* strains had a lipase activity. The presence of DNAses was observed in 45% of the isolated strains of *V. mimicus.* Beshiru [22] reported that 100% of their strains produce DNAses. Pathogens produce nucleases that can degrade extracellular DNA as a mean of escape and spread through tissues. Currently, the majority of *V. vulnificus* and *V. cholerae* strains are positive for DNA destruction [32, 33]. It is known that *V. cholerae* DNAse is secreted to the surrounding environment [34].

### 3.1. Detection of ctxA, tcpA, and toxR Genes

Fragments of the *ctxA* and *tcpA* genes could not be amplified in any of the studied strains of *V. mimicus* (Table 3). The absence of these two genetic elements is consistent with the report from Shinoda [7], which indicated that the presence of a phage in *V. mimicus* strains of environmental origin is lower than 1% [20, 34]. There are *V. mimicus* strains, in which VPI1 is incomplete; these strains lack the gene that encodes the structural protein of the TCP pilus *(tcpA*) or do not possess the *toxT* regulator gene [7, 24].

**Figure 2:**
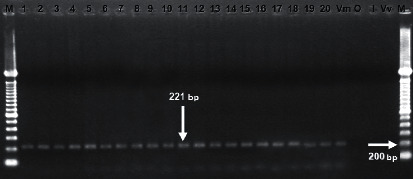
Electrophoresis gel of *toxR* gene amplification product. Lanes (M): 100 bp molecular weight marker; lane 1: *V. mimicus* ATCC 33653; lanes 2–20: isolated strains of *V. mimicus*; lane (O): *V. cholerae* O1 Ogawa; lane (I): *V. cholerae* O1 Inaba; lane (Vv): *V. vulnificus* ATCC 29307.

In 100% of the studied strains, the *toxR* gene was amplified (Table 3) (Figure 2). Shinoda [7] and Provenzano [35] indicated that this gene is present in all species of the *Vibrio* genus. This gene encodes a transmembrane protein that regulates many of the virulence functions in *V. mimicus* and *V. cholerae*; its presence allows *V. mimicus* to regulate its own virulence functions and change the external membrane protein expression pattern in response to environmental stimuli, favoring intestinal colonization, hemolysin expression and flagella mobility [36]. This protein is capable of regulating genes that are present in the pathogenicity island and those of the phage [17].

### 3.2. Siderophores

**Figure 3:**
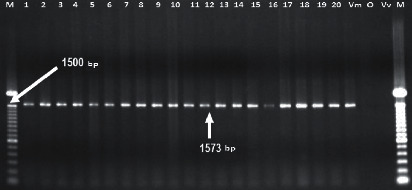
Electrophoresis gel of *iuT* gene amplification product. Lanes (M): 100 bp molecular weight marker; lanes 1–20: isolated strains of *V. mimicus*; lane (Vm): *V. mimicus* ATCC 33653; lane (O) *V. cholerae* O1 Ogawa; lane (Vv): *V. vulnificus* ATCC 29307.

A total of 40% of the strains were capable of producing hydroxamate-type siderophores in the CAS agar, and we found that all of the working strains contain the *iutT* gene (Table 3) (Figure 3). It has been reported that *V. mimicus* produces aerobactin in response to iron deprivation, and the operon *iucABCD iutA* is involved in the synthesis of this hydroxamate-type siderophore [37].

Even though we found *iutT* gene in 100% of the strains, not all of them were capable of producing siderophores. The toxicity of some components in the CAS medium has been reported and can inhibit the growth of some microorganisms [38]. There are not enough reports about the prevalence of *iutT* gene in *V. mimicus*; nevertheless, Moon [39] found the aerobactin operon in the clinical and environmental *V. mimicus* strains. Besides, these authors suggest that these genes are located on the bacterial chromosome and are widely distributed among strains of this species.

It is worth mentioning that the role of siderophores as virulence factors is questioned in other species such as *Klebsiella pneumoniae*, *Shigella* spp., and invasive *Escherichia coli* that lost their pathogenicity when the aerobactin operon is lost. This fact shows that the iron intake mechanisms are a key factor in the process of host infection [40].

### 3.3. Effect of the Cell-Free Filtrates on the CHO Cell Line

**Figure 4:**
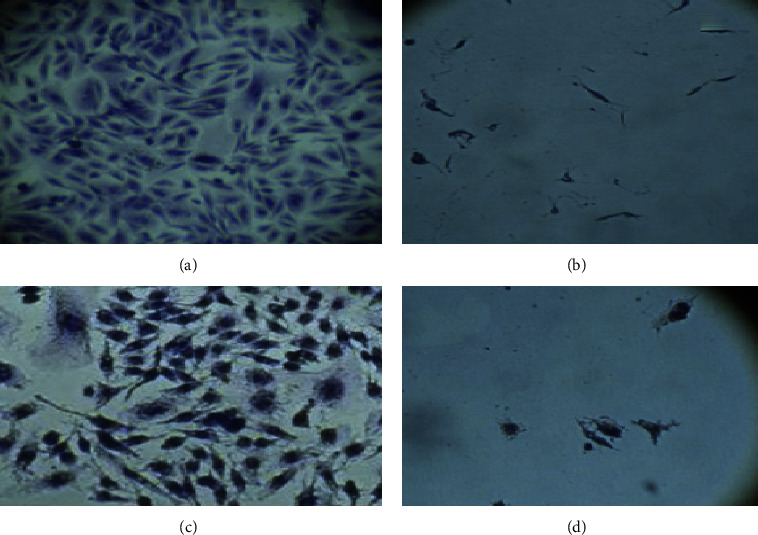
Cytotoxicity assay on CHO cell line. (a) CHO cell control. (b) Positive control with *V. cholerae* O1 serotype Ogawa filtrate. (c) Cytopathic effect caused by *V. mimicus* filtrates after three hours of exposure. (d) Destruction of the cell monolayer caused by the *V. mimicus* filtrates after six hours of exposure (inverted microscope 40X).

Seventy-five percent of all *V. mimicus* strains (15/20) showed a cytopathic effect within 3 h and monolayer destruction after 6 h (Figure 4). The rest (25%) only showed a cytopathic effect characterized by cell loss of structure. The titer of the filtrates of *V. mimicus* ranged from 1 : 3 to 1 : 81, and there is no precedent for estimating their activity. Nevertheless, Baffone [5] found that *V. alginolyticus, V. parahaemolyticus*, and *V. cholerae* no O1 filtrates had titers ranging from 1 : 4 to 1 : 10, considering that the latter strains are highly cytotoxic. Bag [41] reported that isolates of *V. cholerae* no O1/no O139 present titers ranging from 1 : 4 to 1 : 128.

In our study, we found that a titer below 1 : 9 results in a cytopathic phenotype, whereas, with a higher titer, the effect is cytotoxic reaching 1 : 81. The difference among the titers might be caused by variations in the expression of the hemolysins that are the most common cause of destruction and cell damage effect produced by *Vibrio* genus [41].

### 3.4. Adherence Assay

**Figure 5:**
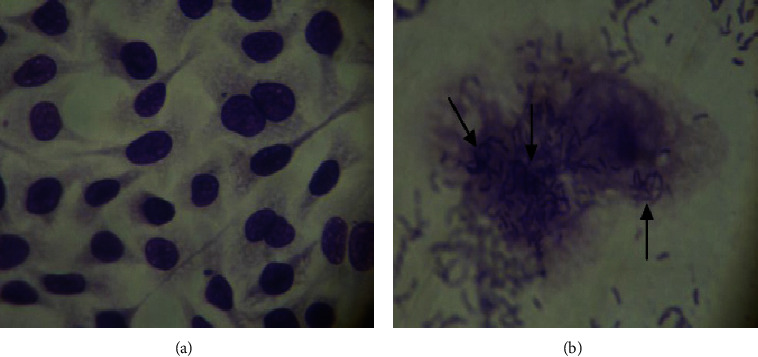
Adherence of *V. mimicus* to HEp-2 cells. (a) Negative control, HEp-2 cells. (b) Adherence pattern exhibited by the *V. mimicus* strains (light microscope 100X).

We found that 100% (20/20) of the strains were adherent (Figure 5). These results concur with previous reports [21, 42, 43], describing *V. mimicus* in a multifactorial adherence process on the surface of intestinal cells in mice [43].

## 4. Conclusions

In this study, we find virulence factors in *V. mimicus* strains as shown for other vibrios. These determinants may enable the microorganism to invade the host and cause tissue damage in order to access nutrient sources required for its growth and propagation. The fishery products contaminated with *V. mimicus* might be a risk if consumed raw or undercooked because it could cause gastroenteritis outbreaks; its ability to adapt to environmental changes and production of different metabolites is what allows it to subsist in the host. This finding leads us to suggest further research to determine the presence of other potential *V. mimicus* virulence factors. Studies on virulence factors in other species of the *Vibrio* genus offer information that could be used to understand the pathogenicity of this bacterium.

## Figures and Tables

**Table 1:** :Primers used in this study.

Target	Primer (5'⟶3′)	Reference
*vmhA*	Fwd GGTAGYCATCAGTCTCATCACG Rev TCRTSTCCCAATACTTCACCG	Present study
*ctxA*	Fwd CTCAGACGGGATTTGTTAGGCACG Rev TCTATCTCTGTAGCCCCTATTACG	[7]
*tcpA*	Fwd GAAGAAGTTTGTAAAAGAAGAACAC Rev GAAAGGACCTTCTTTCACGTTG	[17]
*toxR*	Fwd ACAACAGCGACTCCTCAGAA Rev ACACACAGTTCTATCGAGGG	[7]
*iut*	Fwd AACCGCTACCAAATGACCCCAGAT Rev CAAAACCGGCGACAGAACCTACTT	Present study

**Table 2:** Conditions used for the detection of genes.

Gene	*vmhA*	*ctxA*	*tcpA*	*toxR*	*iut*
Initial denaturation	95°C/5 min	95°C/5 min	95°C/5 min	95°C/5 min	95°C/5 min
Denaturation	95°C/45s	95°C/45s	95°C/45s	95°C/45s	95°C/45s
Annealing	58°C/45 s	55°C/30 s	57°C/40 s	51°C/45 s	62°C/1 min
Extension	72°C/45s	72°C/45s	72°C/45s	72°C/45s	72°C/1 min
Amplicon size (bp)	389	301	472	221	1573

**Table 3:** Genotypic and phenotypic results of working strains.

Strain	Origin	*β*−Hemolysis	*vmh*	Siderophore	*iut*	*ctx*	*tcpA*	*toxR*	Cytotoxicity	Titer of the filtrates	Adherence
Vm	ATCC	+	+	+	+	−	−	+	+	1 : 27	+
1	*W*	+	+	−	+	−	−	+	+	1 : 27	+
2	*W*	+	+	−	+	−	−	+	+	1 : 27	+
3	*O*	+	+	+	+	−	−	+	+	1 : 27	+
4	*O*	+	+	−	+	−	−	+	+	1 : 27	+
5	*F*	+	+	+	+	−	−	+	−	1 : 9	+
6	*F*	+	+	+	+	−	−	+	−	1 : 3	+
7	*F*	+	+	+	+	−	−	+	+	1 : 81	+
8	*W*	+	+	−	+	−	−	+	+	1 : 81	+
9	*W*	+	+	−	+	−	−	+	+	1 : 81	+
10	*W*	+	+	+	+	−	−	+	−	1 : 3	+
11	*W*	+	+	−	+	−	−	+	−	1 : 3	+
12	*O*	+	+	−	+	−	−	+	+	1 : 27	+
13	*O*	+	+	−	+	−	−	+	+	1 : 81	+
14	*O*	+	+	−	+	−	−	+	+	1 : 81	+
15	*O*	+	+	+	+	−	−	+	−	1 : 9	+
16	*O*	+	+	−	+	−	−	+	+	1 : 27	+
17	*F*	+	+	+	+	−	−	+	+	1 : 27	+
18	*O*	+	+	−	+	−	−	+	+	1 : 27	+
19	*O*	+	+	+	+	−	−	+	+	1 : 27	+
20	*O*	+	+	−	+	−	−	+	+	1 : 27	+

*W* = sea water; *O* = oyster; *F*= fish.

## Data Availability

The data used to support the findings of this study are included within the article.
